# Integrative Omics Reveals Rapidly Evolving Regulatory Sequences Driving Primate Brain Evolution

**DOI:** 10.1093/molbev/msad173

**Published:** 2023-07-26

**Authors:** Xiao-Lin Zhuang, Jin-Jin Zhang, Yong Shao, Yaxin Ye, Chun-Yan Chen, Long Zhou, Zheng-bo Wang, Xin Luo, Bing Su, Yong-Gang Yao, David N Cooper, Ben-Xia Hu, Lu Wang, Xiao-Guang Qi, Jiangwei Lin, Guo-Jie Zhang, Wen Wang, Nengyin Sheng, Dong-Dong Wu

**Affiliations:** State Key Laboratory of Genetic Resources and Evolution, Kunming Natural History Museum of Zoology, Kunming Institute of Zoology, Chinese Academy of Sciences, Kunming, Yunnan, China; Kunming College of Life Science, University of the Chinese Academy of Sciences, Kunming, Yunnan, China; State Key Laboratory of Genetic Resources and Evolution, Kunming Natural History Museum of Zoology, Kunming Institute of Zoology, Chinese Academy of Sciences, Kunming, Yunnan, China; Kunming College of Life Science, University of the Chinese Academy of Sciences, Kunming, Yunnan, China; State Key Laboratory of Genetic Resources and Evolution, Kunming Natural History Museum of Zoology, Kunming Institute of Zoology, Chinese Academy of Sciences, Kunming, Yunnan, China; Kunming College of Life Science, University of the Chinese Academy of Sciences, Kunming, Yunnan, China; State Key Laboratory of Genetic Resources and Evolution, Kunming Natural History Museum of Zoology, Kunming Institute of Zoology, Chinese Academy of Sciences, Kunming, Yunnan, China; Kunming College of Life Science, University of the Chinese Academy of Sciences, Kunming, Yunnan, China; School of Ecology and Environment, Northwestern Polytechnical University, Xi’an, Shaanxi, China; Center for Evolutionary & Organismal Biology, Zhejiang University School of Medicine, Hangzhou, Zhejiang, China; Center of Evolutionary & Organismal Biology, and Women’s Hospital at Zhejiang University School of Medicine, Hangzhou, Guangdong, China; Liangzhu Laboratory, Zhejiang University Medical Center, Hangzhou, Guangdong, China; Yunnan Key Laboratory of Primate Biomedicine Research, Institute of Primate Translational Medicine, Kunming University of Science and Technology, Kunming, Yunnan, China; State Key Laboratory of Genetic Resources and Evolution, Kunming Natural History Museum of Zoology, Kunming Institute of Zoology, Chinese Academy of Sciences, Kunming, Yunnan, China; Kunming College of Life Science, University of the Chinese Academy of Sciences, Kunming, Yunnan, China; State Key Laboratory of Genetic Resources and Evolution, Kunming Natural History Museum of Zoology, Kunming Institute of Zoology, Chinese Academy of Sciences, Kunming, Yunnan, China; Center for Excellence in Animal Evolution and Genetics, Chinese Academy of Sciences, Kunming, Yunnan, China; Kunming College of Life Science, University of the Chinese Academy of Sciences, Kunming, Yunnan, China; Key Laboratory of Animal Models and Human Disease Mechanisms of Chinese Academy of Sciences & Yunnan Province, Kunming Institute of Zoology, Chinese Academy of Sciences, Kunming, Yunnan, China; National Resource Center for Non-Human Primates, Kunming Primate Research Center, and National Research Facility for Phenotypic & Genetic Analysis of Model Animals (Primate Facility), Kunming Institute of Zoology, Chinese Academy of Sciences, Kunming, Yunnan, China; KIZ-CUHK Joint Laboratory of Bioresources and Molecular Research in Common Diseases, Kunming Institute of Zoology, Chinese Academy of Sciences, Kunming, Yunnan, China; Institute of Medical Genetics, School of Medicine, Cardiff University, Cardiff, Wales, UK; State Key Laboratory of Genetic Resources and Evolution, Kunming Natural History Museum of Zoology, Kunming Institute of Zoology, Chinese Academy of Sciences, Kunming, Yunnan, China; College of Life Sciences, Northwest University, Xi’an, Shaanxi, China; College of Life Sciences, Northwest University, Xi’an, Shaanxi, China; State Key Laboratory of Genetic Resources and Evolution, Kunming Natural History Museum of Zoology, Kunming Institute of Zoology, Chinese Academy of Sciences, Kunming, Yunnan, China; Center of Evolutionary & Organismal Biology, and Women’s Hospital at Zhejiang University School of Medicine, Hangzhou, Guangdong, China; Liangzhu Laboratory, Zhejiang University Medical Center, Hangzhou, Guangdong, China; State Key Laboratory of Genetic Resources and Evolution, Kunming Natural History Museum of Zoology, Kunming Institute of Zoology, Chinese Academy of Sciences, Kunming, Yunnan, China; School of Ecology and Environment, Northwestern Polytechnical University, Xi’an, Shaanxi, China; Center for Excellence in Animal Evolution and Genetics, Chinese Academy of Sciences, Kunming, Yunnan, China; State Key Laboratory of Genetic Resources and Evolution, Kunming Natural History Museum of Zoology, Kunming Institute of Zoology, Chinese Academy of Sciences, Kunming, Yunnan, China; Center for Excellence in Animal Evolution and Genetics, Chinese Academy of Sciences, Kunming, Yunnan, China; State Key Laboratory of Genetic Resources and Evolution, Kunming Natural History Museum of Zoology, Kunming Institute of Zoology, Chinese Academy of Sciences, Kunming, Yunnan, China; Center for Excellence in Animal Evolution and Genetics, Chinese Academy of Sciences, Kunming, Yunnan, China; National Resource Center for Non-Human Primates, Kunming Primate Research Center, and National Research Facility for Phenotypic & Genetic Analysis of Model Animals (Primate Facility), Kunming Institute of Zoology, Chinese Academy of Sciences, Kunming, Yunnan, China; KIZ-CUHK Joint Laboratory of Bioresources and Molecular Research in Common Diseases, Kunming Institute of Zoology, Chinese Academy of Sciences, Kunming, Yunnan, China

**Keywords:** primate, brain, evolution, regulatory sequences, omics

## Abstract

Although the continual expansion of the brain during primate evolution accounts for our enhanced cognitive capabilities, the drivers of brain evolution have scarcely been explored in these ancestral nodes. Here, we performed large-scale comparative genomic, transcriptomic, and epigenomic analyses to investigate the evolutionary alterations acquired by brain genes and provide comprehensive listings of innovatory genetic elements along the evolutionary path from ancestral primates to human. The regulatory sequences associated with brain-expressed genes experienced rapid change, particularly in the ancestor of the Simiiformes. Extensive comparisons of single-cell and bulk transcriptomic data between primate and nonprimate brains revealed that these regulatory sequences may drive the high expression of certain genes in primate brains. Employing in utero electroporation into mouse embryonic cortex, we show that the primate-specific brain-biased gene *BMP7* was recruited, probably in the ancestor of the Simiiformes, to regulate neuronal proliferation in the primate ventricular zone. Our study provides a comprehensive listing of genes and regulatory changes along the brain evolution lineage of ancestral primates leading to human. These data should be invaluable for future functional studies that will deepen our understanding not only of the genetic basis of human brain evolution but also of inherited disease.

## Introduction

The most distinctive characteristic of primates is perhaps their complex brain and enhanced cognitive abilities. Understanding the genetic foundations for the evolution of the primate brain is a central question in biology. Since King and Wilson (1975) first postulated nearly 50 years ago that the phenotypic differences between human and chimpanzee were driven mainly by changes in gene regulation, numerous studies have shown that evolutionary changes in *cis*-regulatory elements, such as promoters and enhancers, played crucial roles in the evolution of the human brain (Pollard, Salama, King et al. 2006; Pollard, Salama, Lambert et al. 2006; Prabhakar et al. 2006; Boyd et al. 2015; Vermunt et al. 2016; Jeong et al. 2021). Over the past decades, studies of comparative multiomics across primate species have yielded many new insights into the genetic underpinnings of human brain evolution (Bernard et al. 2012; Dehay et al. 2015; Bakken et al. 2016; He et al. 2017; Amiri et al. 2018; Uebbing et al. 2021). However, nearly all these efforts have focused on human by comparison with other nonhuman primates. Although the human brain has undergone a star-like process of evolution in terms of its relative volume and increased complexity through the expansion of cortical areas and an increase in density of cortical networks (Dehay et al. 2015), increases in brain volume and complexity may be regarded as a continuing process along a trajectory from primitive primates (e.g., lemur, loris, and tarsier) to the Simiiformes (i.e., monkeys and apes, including humans). As such, we envisage that exploring genomic changes at each ancestral node, particularly the primitive nodes, will expand our insights into the processes that triggered the evolution of primate and human brains. Unfortunately, our knowledge of the genetic structure of these ancestral nodes is still quite limited owing to the limited genomic and brain gene expression data that have been made available.

**Fig. 1.**
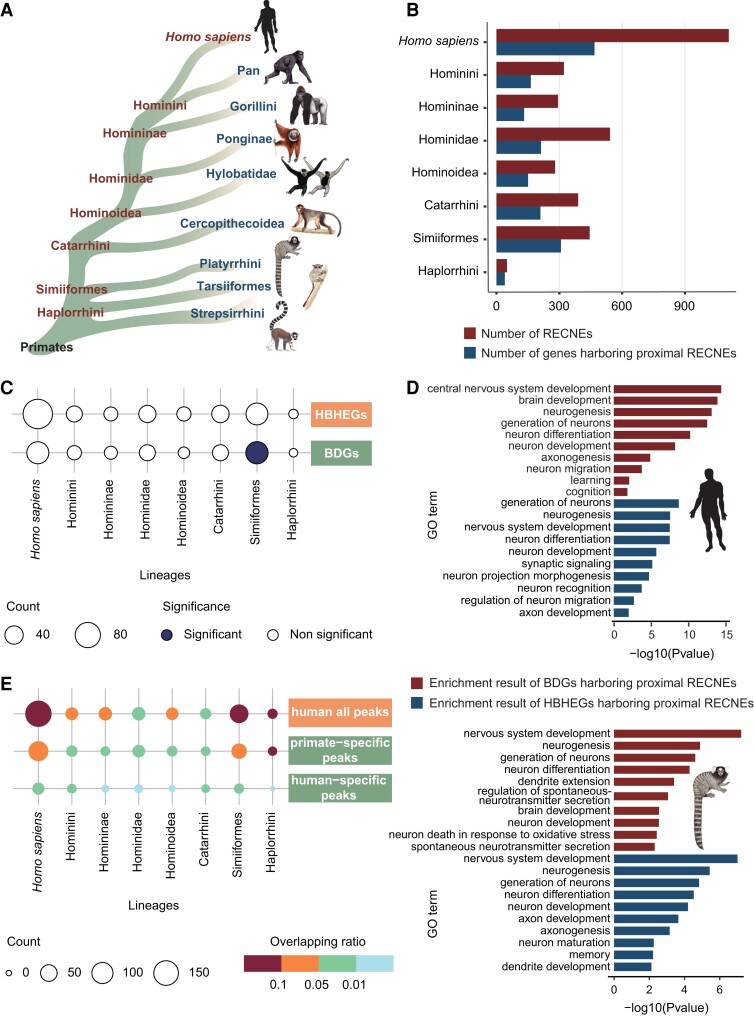
Evolutionary changes of regulatory sequences associated with brain development in the primate lineages. (*A*) Simplified diagram of the phylogenies among different primate lineages. (*B*) The number of RECNEs in each ancestral lineage leading to human (dark red) and the number of genes harboring proximal RECNEs within 500 kb in each ancestral lineage leading to human (dark blue). (*C*) The overlap results between genes related to primate brain development (brain development–related genes [BDGs] and human brain significantly highly expressed genes [HBHEGs]) and genes harboring proximal RECNEs in each primate lineage, respectively. The significance of overlap was measured by the Fisher exact test, and the *P* values were corrected for multiple testing using Benjamini–Hochberg. Counts of overlapping genes were plotted (with the number indicated by dot size), the significance of overlap being denoted by color (dark blue, significant; white, nonsignificant). (*D*) Functional enrichment of BDGs harboring proximal RECNEs (dark red) and HBHEGs harboring proximal RECNEs (dark blue) in the lineages of human (above) and simian ancestor (below). GO enrichments were adjusted for multiple comparisons (FDR < 0.05). (*E*) The overlap results between RECNEs in each primate lineage and activated peaks in human fetal brain, only in primate fetal brain (primate: human and rhesus macaque; compared with mouse), and only in human fetal brain (compared with rhesus macaque and mouse), respectively. Counts of overlapping regions were plotted (with the number indicated by dot size), the ratio of overlap being denoted by color (the ratio of intersection over the total number of RECNEs in each primate lineage was used to depict the extent of overlap).

By comparing the entire genomes of 50 primate species obtained from the Primate Genome Project (Shao et al. 2023), we identified conserved noncoding elements that exhibited rapid evolution (RECNEs; “rapid evolution” refers to the elevated substitution rate of the noncoding sequences in particular lineages across the genome), in all the ancestral primate nodes leading to humans (fig. 1*A*). We further performed phylogenomic analyses with epigenomic data (i.e., histone H3K27ac and H3K4me2 Chip-seq data) of various developmental stages for the brains of human (7 postconception weeks [pcw], 8.5 pcw, and 12 pcw), rhesus macaque (8 pcw and 12 pcw), and mouse (embryonic day 11 [E11], E14, and E17) (Reilly et al. 2015), bulk transcriptome of 72 brain tissues and 96 nonbrain tissues, and >100,000 single-cell transcriptomes of fetal brains from human, rhesus macaques, and tree shrew (*Tupaia belangeri*, representing a close outgroup of primates). By integrating the analysis results from comparative genomics and transcriptomics, we provide a comprehensive list of genetic elements that have potential functions associated with brain evolution at the key evolutionary nodes, including *Homo sapiens* and the ancestor of Simiiformes. We also perform various experiments to validate the functions of some of these key genetic innovations in primate brain development.

## Results and Discussion

### Pronounced Rapid Evolution of Regulatory Sequences in the Ancestor of Simiiformes

Previous research has shown that the evolutionary changes of conserved noncoding sequences may have contributed to the formation of the unique human traits that distinguish humans from other primates (Prabhakar et al. 2006). To comprehensively explore the important role of conserved noncoding sequences in primate evolution, we utilized 50 high-quality primate genomes and the tree shrew (*T. belangeri*, representing a close outgroup of primates) genome from the Primate Genome Project (Shao et al. 2023) and then performed comparative phylogenomic analysis to identify RECNEs in major primate evolutionary nodes with the phyloP program in PHAST (fig. 1*A*, the significant acceleration at false discovery rate [FDR]–adjusted *P* ≤ 0.05). We observed a much higher number of RECNEs in the lineages of *H. sapiens* and the ancestor of the Simiiformes as compared with other primate lineages, and in these two lineages, we detected the highest number of genes harboring proximal RECNEs (fig. 1*B*); these genes in particular are significantly enriched in nervous system–related categories (supplementary fig. S1, Supplementary Material online). Moreover, a markedly increased number of genes that are potentially regulated by RECNEs in the lineages of *H. sapiens* and the ancestor of Simiiformes were highly expressed in the human brain and exhibited a marked association with brain development (fig. 1*C*, supplementary tables S1 and S2, Supplementary Material online); Gene Ontology (GO) enrichment analyses suggested that these genes were overrepresented in nervous system development, especially in “neurogenesis” (GO: 0022008), “neuron differentiation” (GO: 0030182), “neuron development” (GO: 0048666), and “axonogenesis” (GO: 0007409) (fig. 1*D*). These functional pathways represent key stages in the formation of the brain cortex and hence may be important influences on brain volume. It is interesting that the Simiiformes node accumulated a higher number of RECNEs flanking the brain-related genes, which may be related to brain evolution, such as the expansion of brain volume at this key node (Shao et al. 2023).

In addition to the rapid evolution of conserved noncoding elements, newly originated species-specific regulatory sequences also played roles in primate brain evolution. By comparing the epigenomic data (i.e., histone H3K27ac and H3K4me2 Chip-seq data that mark regulatory elements) from brains of human, rhesus macaque, and mouse at different developmental time points (Reilly et al. 2015), we identified 26,535 human-specific (the epigenetic peaks present in 16 fetal human samples but absent in 6 fetal rhesus macaque and 16 fetal mouse samples) and 93,371 primate-specific (the epigenetic peaks present in 16 fetal human and 6 fetal rhesus macaque samples but absent in 16 fetal mouse samples) epigenetic peaks that were likely to be newly originated regulatory elements important for primate brain development in the human lineage or the common ancestor of primates. By overlapping the RECNEs, we found that a higher number of RECNEs in *H. sapiens* and the ancestor of Simiiformes overlapped with human-all, human-specific, and primate-specific epigenetic peaks (fig. 1*E*, supplementary tables S3–S5, Supplementary Material online).

In conclusion, the above results recapitulated the rapid evolution of regulatory sequences associated with brain development in the human lineage, as has been postulated in many previous studies (King and Wilson 1975; Pollard, Salama, King et al. 2006; Prabhakar et al. 2006; Bird et al. 2007; Boyd et al. 2015; Gittelman et al. 2015; Reilly et al. 2015; Dong et al. 2016; Won et al. 2019; Agoglia et al. 2021). It is remarkable that the rapid evolution of the regulatory sequences of brain genes also appeared in the Simiiformes ancestor, a pattern that has not previously been reported. Indeed, the relative brain volume increased significantly in the Simiiformes by comparison with the more “primitive” primates, such as lemur and loris (Shao et al. 2023). It supports the view that brain expansion is a continuing evolutionary trend that has operated from the time of the early primates, rather than simply occurring in the human lineage.

### Single-Cell RNA-Sequencing Identified RECNEs Related to Gene Expression Changes in Brain Cells During Primate Evolution

**Fig. 2.**
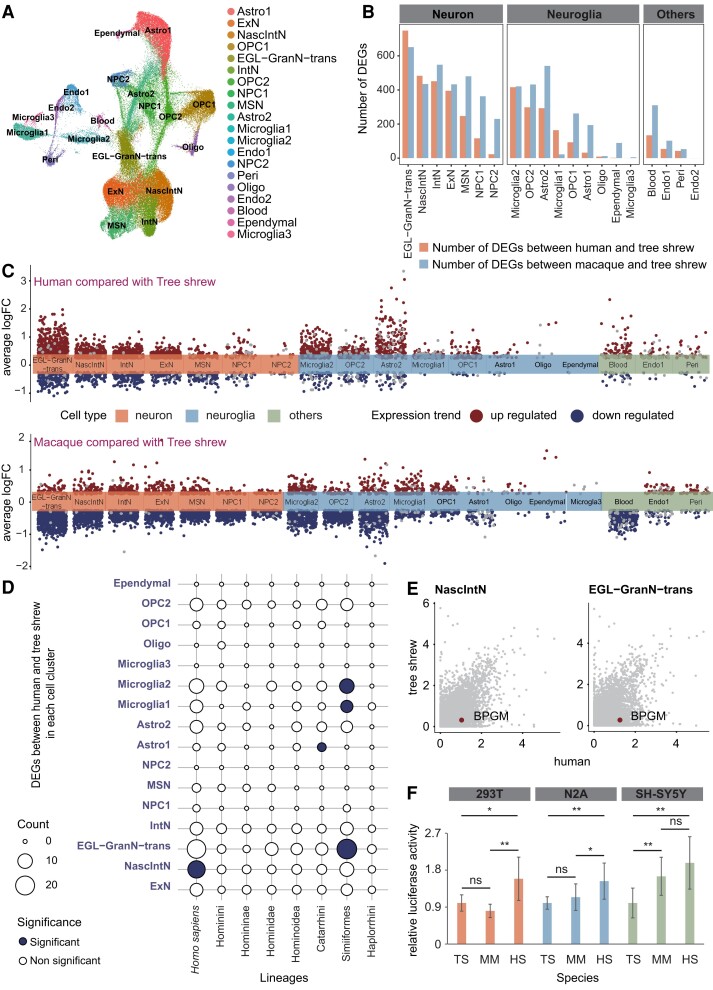
Cellular comparison between primates (human and rhesus macaque) and a nonprimate (tree shrew) using single-cell RNA-sequencing data. (*A*) UMAP plot of single cells from the integrated analysis of three species, labeled by cell type. The full names of the cell types are shown below. (*B*) The number of DEGs between human and tree shrew (orange) and the number of DEGs between rhesus macaque and tree shrew (blue) in each integrated cluster. (*C*) Volcano plot represents the upregulated DEGs (dark red) and downregulated DEGs (dark blue) that compared tree shrew with human (above) and rhesus macaque (below) in each integrated cluster, respectively. (*D*) The overlap results between genes harboring proximal RECNEs in each primate lineage and DEGs between human and tree shrew. The significance of overlap was measured by Fisher's exact test, and the *P* values were corrected for multiple testing using Benjamini–Hochberg. Counts of overlapping genes were plotted (with the number indicated by dot size), the significance of overlap being denoted by color (dark blue, significant; white, nonsignificant). (*E*) Visualization of the expression levels of the gene *BPGM* in human and tree shrew in NascIntN and EGL-GranN-trans. (*F*) Luciferase reporter analyses of the regulatory activity of RECNE for *BPGM* of human (HS), rhesus macaque (MM), and tree shrew (TS). Bar graphs, overlaid with the actual data points, show normalized regulatory activities (mean ± SEM). Every two groups of data were analyzed using an unpaired *t*-test with Welch's correction. *P < 0.05; **P < 0.01; ns *P* > 0.05. NPC, neuronal progenitor cell; NascIntN, nascent inhibitory neuron; ExN, excitatory neuron; Astro, astrocyte; OPC, oligodendrocyte progenitor cell; Oligo, oligodendrocyte; Endo, endothelial; Peri, pericyte; EGL-GranN-trans, external granular layer transformed granule neuron; IntN, inhibitory neuron; MSN, medium spiny neuron.

It is reasonable to suppose that the rapid evolution of regulatory sequences would be accompanied by changes in gene expression. To further explore the functional consequences of RECNEs for primate brain development and evolution, we newly generated single-cell transcriptomes of 28,167 cells from two fetal whole brains of tree shrew by Illumina 10X program and compared them with single-cell transcriptomes from fetal brains of rhesus macaque (including the amygdala, striatum, hippocampus, mediodorsal nucleus of the thalamus, neocortex, and cerebellar cortex) (Zhu et al. 2018) and human (including 20 major anatomical cortical regions, medulla, and pons) (Fan et al. 2018). In total, we identified 20 cell clusters that may be involved in 14 cell types in the brains of the three species by integrated analysis (fig. 2*A*, supplementary fig. S2*A* and *B* and table S6, Supplementary Material online). Differentially expressed genes (DEGs) between human and tree shrew or between rhesus macaque and tree shrew in each cell cluster were identified using the Wilcoxon rank sum test (adjusted *P* < 0.05, fig. 2*B* and *C*, supplementary tables S7 and S8, Supplementary Material online). We found that the external granular layer transformed granule neuron (EGL-GranN-trans) (Zhu et al. 2018) differed most between primates (human and rhesus macaque) and nonprimate (tree shrew) (fig. 2*B* and *C*). When we then performed the GO enrichment analysis for the DEGs between primates (human and rhesus macaque) and nonprimate (tree shrew) in EGL-GranN-trans, the results indicated that these DEGs were essential for brain development (supplementary fig. S3, Supplementary Material online). Further, we found that 17.9% and 42.5% of DEGs between primates (human and rhesus macaque) and nonprimate (tree shrew) could be driven by human-specific and primate-specific regulatory elements, which were defined as newly originated regulatory elements in the human lineage or the common ancestor of primates, identified by H3K27ac and H3K4me2 (supplementary fig. S4*A* and *B* and tables S9 and S10, Supplementary Material online). These results implied that the rapid evolution of regulatory sequences could have modulated the gene expression changes in brain cells during primate evolution.

In particular, the genes harboring RECNEs in the Simiiformes ancestor were found to significantly overlap with DEGs between human and tree shrew in EGL-GranN-trans and microglia cells and significantly overlapped with DEGs between macaque and tree shrew in oligodendrocyte progenitor cells (fig. 2*D*, supplementary fig. S5 and tables S11 and S12, Supplementary Material online); further, the genes harboring RECNEs in the human lineage exhibited a significant overlap with DEGs between human and tree shrew in nascent inhibitory neurons (NascIntN) (fig. 2*D*, supplementary table S11, Supplementary Material online). This pattern corroborated the above finding of rapid evolution of regulatory sequences associated with the brain in the ancestor of the Simiiformes.

Among the RECNEs occurring in the different ancestral lineages leading to human, the key evolutionary nodes *H. sapiens*, Hominidae, Hominoidea, Catarrhini, Simiiformes, and Haplorrhini had respectively 16, 2, 3, 3, 17, and 2 RECNEs linked with genes displaying differential expression levels between human and tree shrew brain cells (supplementary table S11, Supplementary Material online), and these RECNEs showed regulatory activity supported by human fetal brain Chip-seq data (Reilly et al. 2015) (supplementary table S3, Supplementary Material online). For example, one of the top RECNEs in the human lineage (chr7: 134695356-134695576) is located ∼15 kb downstream of the gene *BPGM* which exhibited significantly higher expression levels in neuronal cell types (e.g., NascIntN and EGL-GranN-trans) of human than those of tree shrew (fig. 2*E*, supplementary tables S7 and S11, Supplementary Material online). The regulatory activity of this element in the brain was supported by H3K27ac and H3K4me2 Chip-seq data of the developing human brain (Reilly et al. 2015) (supplementary table S3, Supplementary Material online) and evolved rapidly in the human lineage compared with other primate lineages as detected by the PHAST program. We further employed a luciferase assay to show that during the evolutionary progression from tree shrews to humans, the regulatory activity of this RECNE continued to increase (fig. 2*F*).

### Newly Originated Regulatory Sequences Modifying the Specific Expression Pattern of the Primate Brain

**Fig. 3.**
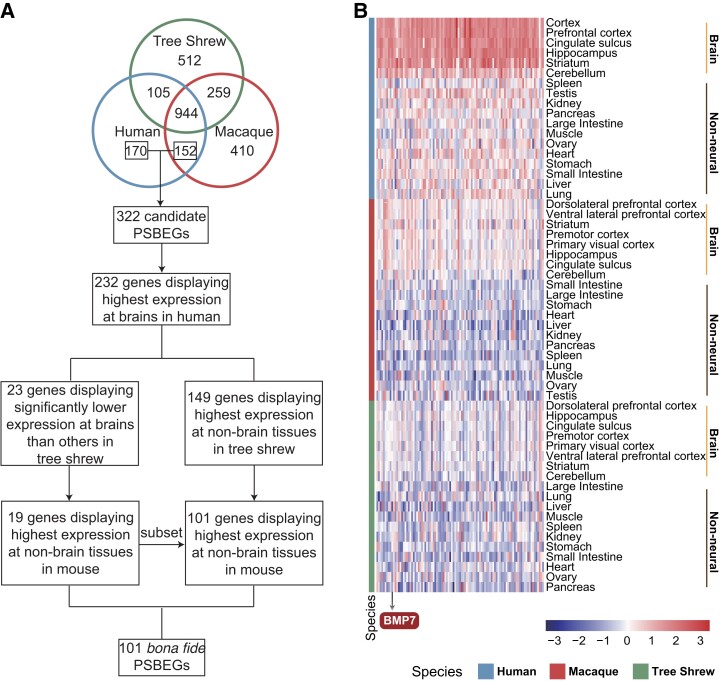
Identification of the PSBEGs. (*A*) The detailed pipeline for identification of the PSBEGs. (*B*) Heatmap depicting normalized expression of 101 PSBEGs in brain and nonneural tissues from human, rhesus macaque, and tree shrew, respectively. Species are labeled by color (blue, human; red, macaque; green, tree shrew). Brain and nonneural tissues are separated in each species (right). Color scales indicate the normalized expression level of the PSBEGs. Gene *BMP7* mentioned in the text is indicated at the bottom.

Besides single-cell transcriptome data, we further generated 84 bulk transcriptomes of the brain and nonbrain tissues from rhesus macaque and tree shrew (supplementary tables S13–S15, Supplementary Material online) and combined these with the transcriptome data of the brain and nonbrain tissues of human and rhesus macaque from previous published studies (Li et al. 2019; Aguet et al. 2020) (supplementary tables S13–S15, Supplementary Material online). Based on these comprehensive transcriptome data sets, we used a stringent pipeline to identify 101 primate-specific brain-biased genes (PSBEGs) which were defined as genes displaying a statistically higher level of expression in the brain than other nonbrain tissues in primates (human and rhesus macaque) but a statistically lower level of expression in the brain as compared with other nonbrain tissues in nonprimates (tree shrew and mouse) (fig. 3*A* and *B*, supplementary figs. S6 and S7 and table S16, Supplementary Material online).

**Fig. 4.**
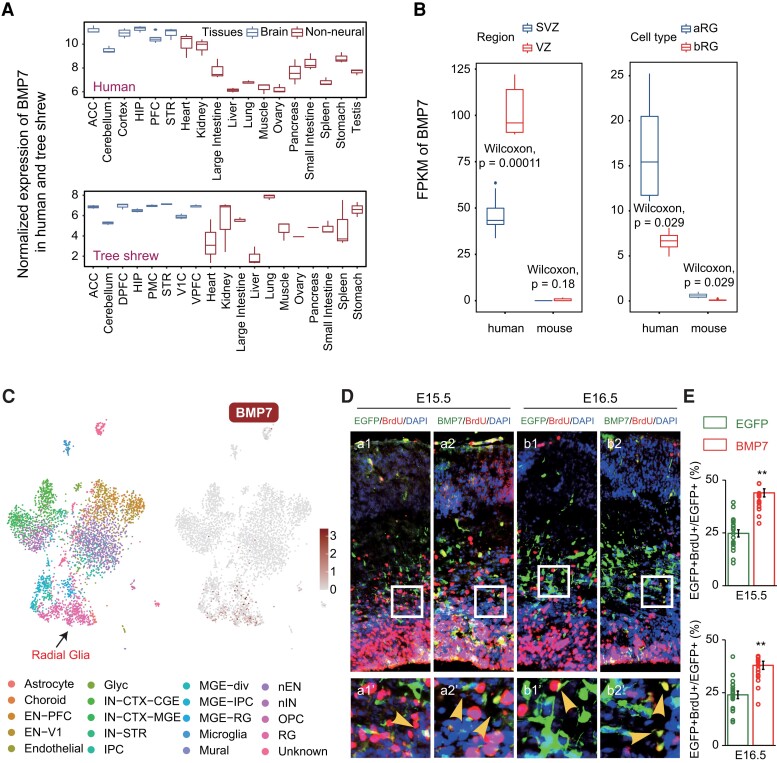
Expression and function of the gene *BMP7*. (*A*) Boxplot depicting the log_2_ transformed normalized expression of *BMP7* in the brain and nonneural tissues from human (top) and tree shrew (bottom). The normalized expression of *BMP7* was calculated by the median of ratios method of DEseq2 (Love et al. 2014); once the sequencing depth and RNA composition had been taken into account, the normalized gene counts could be compared between samples. Tissues are distinguished by color (blue, brain tissues; red, nonneural tissues). The full names of the tissues are shown below. (*B*) Boxplot depicting the expression profile of *BMP7* in SVZ and VZ brain regions (left) and aRG and bRG cells (right) from human and mouse, respectively; transcriptome data were from Fietz et al. (2012) and Florio et al. (2015). *P* values calculated by means of the Wilcoxon test are shown. Regions and cell types are distinguished by color (blue, SVZ, aRG; red, VZ, bRG). (*C*) Single-cell profile of human embryo brain cortex (left) and expression profile of *BMP7* (right). Radial glia are labeled in single-cell profile. The single-cell data of human embryo brain cortex and full names of the cell types were from Nowakowski et al. (2017). (*D*) Coronal brain sections of single EGFP (a1–b1) or combined EGFP/*BMP7* (a2–b2) electroporation at indicated time points were stained with BrdU antibody, as well as DAPI. The number of EGFP/BrdU double-positive cells, as well as EGFP single-positive cells, was then counted. Expanded images at the bottom of each panel are taken from the area indicated by white rectangles. The yellow arrowheads in the expanded images pointed at EGFP^+^BrdU^+^ cells. Scale bar: 50 μm (original) and 12.5 μm (enlarged). (*E*) Quantification of EGFP^+^BrdU^+^ cells to respective total EGFP^+^ cells of indicated treatment at different time points in figure 3*E*. The quantification was performed in the areas indicated by white rectangles within the images as shown in (*D*). Bar graphs, overlaid with the actual data points, show normalized ratios (mean ± SEM). Data were analyzed using an unpaired *t*-test with Welch's correction. **0.001 < *P* < 0.01. ACC, anterior cingulate cortex; HIP, hippocampus; PFC, frontal cortex; STR, striatum; DPFC, dorsolateral prefrontal cortex; PMC, premotor cortex; V1C, primary visual cortex; VPFC, ventral lateral prefrontal cortex.

To identify the PSBEGs that played important roles in primate brain during early development, we further focused on the subventricular zone (SVZ) and ventricular zone (VZ) of the neocortex, as well as the apical radial glia (aRG) and basal radial glia (bRG) cells in fetal human and fetal mouse, considering that the expansion and increase in complexity of the human neocortex were mainly attributable to the expansion of the SVZ, which resulted primarily from the increased amplification of basal progenitors, that is, bRG, leading to the increased production of neurons during fetal corticogenesis (Fietz et al. 2012; Florio et al. 2015). Among these PSBEGs, three genes (bone morphogenetic protein 7 [*BMP7*; ENSG00000101144], *DIO2* [ENSG00000211448], and *COL9A1* [ENSG00000112280]) displayed higher expression levels in the SVZ, VZ, aRG, and bRG of fetal human but lower expression levels in fetal mouse [log_2_ (FPKM + 1) greater than 1 in more than 80% samples of fetal human and log_2_ (FPKM + 1) less than 1 in more than 80% samples of fetal mouse, supplementary figure S8*A*–*C*, Supplementary Material online]. *BMP7* encodes a secreted ligand of the transforming growth factor-beta (TGF-beta) superfamily of proteins, which could contribute to bone, kidney, and brown adipose tissue development. Additionally, one previous study evidenced potential functions for *Bmp7* in murine corticogenesis (Segklia et al. 2012). In particular, PSBEG *BMP7* (fig. 4*A*) displayed a statistically higher level of expression in the VZ and SVZ of the human fetus (Fietz et al. 2012) as well as very high expression levels in fetal human aRG and bRG (Florio et al. 2015) (fig. 4*B*). By contrast, there was nearly no *Bmp7* expression in the fetal brain of mouse (Fietz et al. 2012; Florio et al. 2015) (fig. 4*B*). The single-cell RNA-sequencing data from human brain during early developmental stages provided further support for the expression of *BMP7* in radial glia (Nowakowski et al. 2017) (fig. 4*C*), but not in embryonic mouse brain (Li et al. 2020), corresponding to the results in figure 4*B*. To ascertain the function of *BMP7* in human neural stem cells (NSCs)/neural progenitor cells (NPCs) in the VZ/SVZ region during early brain development, we used in utero electroporation (IUE) (supplementary fig. S9, Supplementary Material online) to ectopically express the human *BMP7* gene together with the enhanced green fluorescent protein (*EGFP*) reporter gene in mouse embryonic cortex around E13.5, a stage when endogenous *Bmp7* expression was barely detectable. The development of both human and mouse brains all originated primarily from an increase in NSCs/NPCs in the VZ/SVZ (Fietz et al. 2012). Thus, the experimental results in mouse embryos could reflect the function of gene *BMP7* in human embryos. We noted a statistically significant increase in the ratio of BrdU-positive cells (BrdU: 5-bromo-2'-deoxyuridine) in *BMP7*-overexpressed embryos as compared with control *EGFP*-only ones (data were analyzed using an unpaired *t*-test with Welch's correction, fig. 4*D* and *E*) in E15.5 and E16.5, suggesting that *BMP7* expression in the human embryonic VZ/SVZ could promote the proliferative ability of NSCs/NPCs, which could in turn have facilitated brain expansion in primates.

**Fig. 5.**
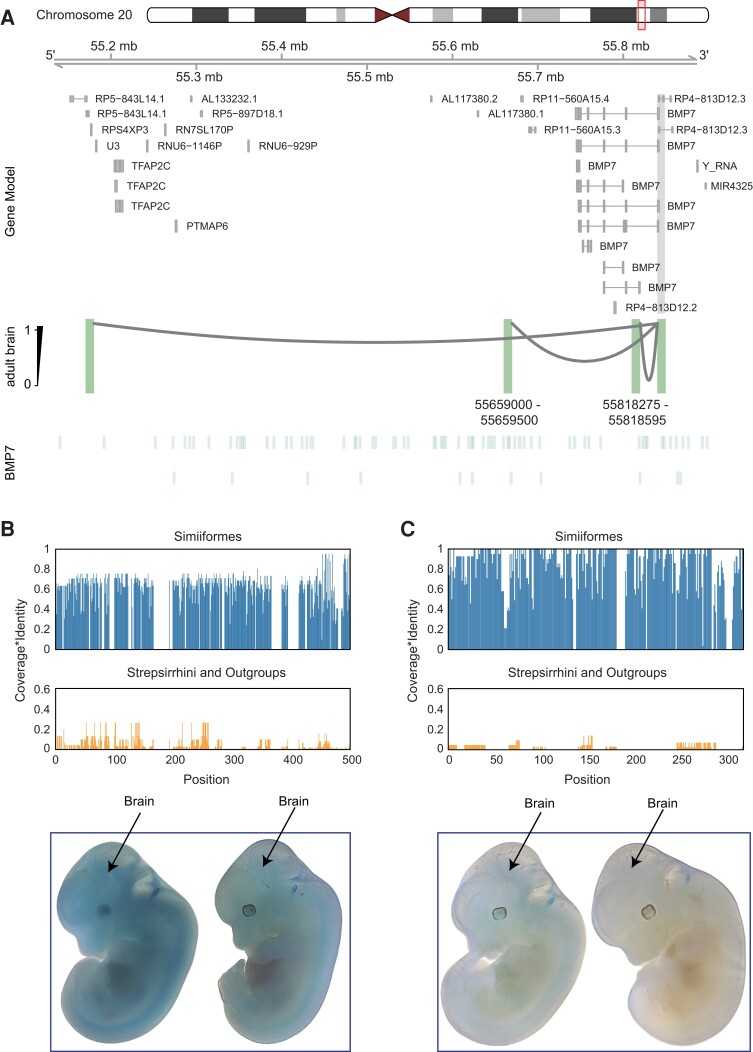
Origin of *BMP7* regulatory sequences. (*A*) In human adult brain, the *BMP7* gene interacted with distal *cis*-regulatory elements via loops detected by Hi-C data (Wang et al. 2018). The regions that interact with the gene promoter (filled in gray) are highlighted in green. The coordinates (reference genome: hg19) of two human specifically activated promoters or enhancers around the *BMP7* gene are shown below. (*B*, *C*) Conservation of the two regulatory elements surrounding the *BMP7* gene (*B*, hg19 coordinates: chr20: 55659000-55659500; *C*, hg19 coordinates: chr20: 55818275-55818595) in Simiiformes, Strepsirrhini, and outgroups. The *x*-axis is the sequence position inside the two regulatory elements, whereas the *y*-axis is the product of base coverage and base alignment similarity. Below are representative E11.5 transgenic embryos obtained for two regulatory elements. The brain regions in each embryo were indicated by arrows. Each construct shows at least three embryos resulting from independent transgene integration events.

When searching for regulatory changes underlying these PSBEGs, we found that only a few PSBEGs contain RECNEs (16.83%, supplementary fig. S10*A* and table S17, Supplementary Material online). We reasoned that the evolution of expression of PSBEGs may involve another mechanism, such as newly originated primate-specific or human-specific regulatory sequences. Indeed, the use of chromatin interaction data from developing human, rhesus macaque, and mouse brain tissues allowed us to conclude that many PSBEGs (56.44%, supplementary fig. S10*B* and table S18, Supplementary Material online) may be subject to human-specific or primate-specific epigenetic peaks (Reilly et al. 2015). By comparing the chromatin interaction data of brain tissues between fetal human, fetal macaque, and fetal mouse (Reilly et al. 2015), we discovered that human *BMP7* acquired two regulatory elements that are specifically active in human fetal brain (fig. 5*A*, supplementary fig. S11, Supplementary Material online) and which could have contributed to the specific expression pattern of this gene in the developing human. Multispecies sequence alignment demonstrated high conservation of the two regulatory elements across different species of Simiiformes, but not in the Strepsirrhini (i.e., lemur and loris) and other outgroup species (fig. 5*B* and *C*), suggesting the potential origin of these two elements in the common ancestor of Simiiformes. To further verify the regulatory activities of the two regulatory elements in embryo brains, we performed LacZ transgenic reporter experiments on the two regulatory elements in mouse embryos. Notably, the transgene expression profiles demonstrated that the element (chr20: 55659000-55659500, hg19) showed broad regulatory activity in mouse embryos including brains (fig. 5*B*), but the element (chr20: 55818275-55818595, hg19) had no regulatory activity. We proposed that the element (chr20: 55659000-55659500, hg19) could drive high expression of *BMP7* in primates compared with nonprimates across different tissues, not only in the brain. Indeed, when comparing the expression of *BMP7* between human and tree shrew across different tissues, we found that *BMP7* showed significantly higher expression levels in many human tissues than tree shrew tissues, including different brain regions (supplementary fig. S12, Supplementary Material online). Together, *BMP7* may have been recruited to function in the development of the SVZ and VZ of the neocortex in Simiiformes by acquiring primate-specific regulatory elements, which further corroborates the rapid evolution of the brain in the Simiiformes ancestor described above.

## Conclusions

In this study, our systematic comparative analyses of genomics, transcriptomics, and epigenomics yielded a comprehensive lexicon of genetic elements including rapidly evolving conserved noncoding elements and primate-specific regulatory sequences that have potential functions related to brain evolution in each ancestral primate lineage leading to human. This list of genetic elements is a very promising starting point for future functional studies that could deepen our understanding of the genetic basis of human brain evolution, with important implications for our comprehension of neurological and neuropsychiatric diseases. In particular, our study unveils the functions of the *BMP7* gene in neuronal proliferation, which could have played a role in the expansion of the primate brain. In addition, we note that these innovatory genetic elements occurred frequently at the node of the Simiiformes ancestor, a finding that was previously unappreciated and which is supportive of brain expansion being a continuing evolutionary trend that has operated from the time of the earliest primates, rather than simply occurring in the human lineage. As such, we envisage that future investigations should focus on the ancestral primate lineages, as a means to potentiate the detailed reconstruction of the molecular alterations that triggered primate brain evolution.

## Materials and Methods

### Data Description

Primate brain evolution has been characterized by different primate species undergoing distinct changes on multiple levels. It was therefore necessary to analyze multiple brains from primate species representing major lineages at multiple levels to allow the identification of regulatory/expression changes in the primate brain. This study integrated comparative genomic, transcriptomic, and epigenomic analyses of primates, utilizing huge amounts of genomic, transcriptomic, single-cell transcriptomic, and epigenomic data. Although most data were generated by our laboratory, other data were obtained from previously published sources, as detailed below.

#### Genomes

Fifty primate genomes have been described and analyzed in detail in the accompanying paper (Shao et al. 2023). In this study, we utilized a list of RECNEs from major ancestral lineages leading to human (including *H. sapiens*, Hominini, Homininae, Hominidae, Hominoidea, Catarrhini, Simiiformes, and Haplorrhini) from our accompanying paper (Shao et al. 2023) to perform integrative analyses with transcriptomes and epigenomes. We annotated the nearest gene surrounding each RECNE within 500 kb by using the reference annotation of human genome (*H. sapiens*, hg38, Ensembl v100).

#### Transcriptomes

We used abundant brain and nonbrain transcriptomes of adult individuals from human, rhesus macaque, and tree shrew for identifying PSBEGs (supplementary tables S13–S15, Supplementary Material online). More specifically, 24 brain and 36 nonbrain transcriptomes from human were randomly selected from the GTEx project data (release V8) (Aguet et al. 2020). Simultaneously, 24 brain transcriptomes from rhesus macaques were randomly selected from the data set previously published by our laboratory (Li et al. 2019), and 32 nonbrain transcriptomes from the same individuals that provided brain tissues were newly generated. Moreover, we generated 24 brain and 28 nonbrain transcriptomes from three tree shrews specifically for this study.

#### Single-Cell Transcriptomes

We integrated more than 100,000 single-cell transcriptomes from embryonic human, rhesus macaque, and tree shrew. Human single-cell RNA-sequencing data were obtained from published data, over 4000 individual cells encompassing 22 brain regions (including 20 major anatomical cortical regions, medulla, and pons) of midgestation embryos (22–23 weeks postconception) (Fan et al. 2018). For rhesus macaque, we utilized single-cell RNA-sequencing data from six brain regions (including the amygdala, striatum, hippocampus, mediodorsal nucleus of the thalamus, neocortex, and cerebellar cortex) of two E110 embryos from previously published studies (Zhu et al. 2018). For tree shrew, we newly generated single-cell RNA-sequencing data of whole brain from two two-thirds-gestation embryos (4–5 postconceptional weeks).

#### Epigenomes

We downloaded chromatin interaction data of fetal brains from human (7 pcw, 8.5 pcw, and 12 pcw), rhesus macaque (8 pcw and 12 pcw), and mouse (E11, E14, and E17) from a previously published paper (Reilly et al. 2015).

### Tissue Dissection

Rhesus macaque nonbrain samples were collected from three adult individuals' postmortem, all 6 years old. The three animals were used in our previous study (Li et al. 2019). Tree shrew brain and nonbrain samples were collected from three adult individuals' postmortem. Moreover, whole brains from tree shrew embryos were collected from two two-thirds-gestation embryos (4–5 postconceptional weeks) for single-cell RNA-sequencing.

All individuals with no previously reported disease were obtained from the Kunming Primate Research Center, Chinese Academy of Sciences (AAALAC accredited). The procedure of animal care and use was approved by the Institutional Animal Care and Use Committee (IACUC) at the Kunming Institute of Zoology (approval number: SMKX-20201115-29), Chinese Academy of Sciences.

The tissue samples were dissected and collected by a skilled technician from the Animal Branch of the Germplasm Bank of Wild Species, Chinese Academy of Sciences (the Large Research Infrastructure Funding). All surgical instruments (surgical scissors and tweezers) used for dissection were sterilized in advance and used for each sample only once to avoid cross-contamination. To ensure consistency in sampling between specimens, all dissections were performed by the same person.

After the dissection, 100-mg samples of each tissue were stored in freezing tubes with RNAlater solution (AM7021, Ambion, USA) at liquid nitrogen temperature and then immediately stored at −80 °C. The entire brains of the tree shrew embryos were immediately transferred to ice-cold Dulbecco's Modified Eagle Medium (DMEM) solution with 10% fetal bovine serun (FBS) (DMEM/FBS) after dissection, for preparation of the single-cell suspension.

### Sample Preparation and Sequencing for Single-Cell RNA-Sequencing

#### Single-Cell Dissociation

All operations were performed on ice. Centrifugation of the samples was performed at 4 °C. After transferring the whole brains of tree shrew embryos to ice-cold DMEM/FBS, the dura mater and blood vessels were removed from the brains. Brain tissues were dissociated using a modified protocol from the Worthington Papain Dissociation System. In brief, the dissected brains were cut into small pieces of less than 1 mm^3^. The small pieces were then digested with 1 mg/ml papain and 0.4% DNaseI at 37 °C for 30 min, shaking once every 5 min. After 40 min, the digestion process was blocked with DMEM/FBS. Cell suspensions were filtered through a 100-µm cell strainer and pelleted in a benchtop centrifuge at 200 × g for 5 min. Pellets were resuspended in DMEM and strained through a 40-µm filter.

To remove cell debris, 2 ml cell suspension was layered onto a gradient composed of 1 ml layers of 11%, 20%, and 35% Optiprep1.32, pH 7.4, in DMEM and centrifuged for 5 min at 430 × g with no brake. The bottom 1 ml of the gradient was collected and mixed with 1 ml DMEM. The cells were then pelleted at 550 × g for 5 min before being resuspended in 100–200 µl DMEM with 0.2% bovine albumin. Quality control required cell viability to be higher than 80%. The cells of the brain of each tree shrew embryo were then processed with Chromium Single Cell 3′ Reagent Kits following the manufacturer's instructions (v3 chemistry CG000183).

#### Library Construction and Sequencing

The input cells were loaded onto the channel of Single Cell B Chip (v3 chemistry, PN-1000153). The 10x library was constructed using 10x Chromium Controller and Chromium Single Cell 3′ Reagent Kits (v3 chemistry CG000183) following the detailed protocol of the manufacturer. In brief, single-cell suspensions in each channel of the chip were loaded on a Chromium Controller (10x Genomics, Pleasanton, CA) to generate single-cell GEMs (gel beads in the emulsion). The 10x libraries of each channel were then prepared using the Chromium Single Cell 3′Gel Bead and Library Kit v3 (PN-1000153, 1000075, 120262; 10x Genomics).

Libraries were sequenced using an Illumina NovaSeq 6000 sequencer with 150-bp paired-end reads.

### Single-Cell RNA-Sequencing Data Processing and Analysis

#### Quality Control of Raw Data

We used fastp (Chen et al. 2018) to perform basic statistics on the quality of the raw reads, ensuring the raw reads of Read1 and Read2 were adequate for subsequent analyses. In total, we sequenced 9,241 and 9,685 single cells from two tree shrew embryos, respectively.

#### Reads’ Alignment

The raw data were analyzed by cellranger 3.1.0. In brief, the cellranger mkfastq transformed raw base call (BCL) files generated by Illumina sequencers into FASTQ files and decoded the multiplexed samples. Cellranger count took FASTQ files from cellranger mkfastq and performed sequencing alignment, filtering, barcode counting, and unique molecular identifiers counting using the default parameters. The reference genome and annotation files of the tree shrew were downloaded from the Tree Shrew Database (TS_2.0 long-read assembly, http://www.treeshrewdb.org/). The standard output files generated by cellranger count served as input data for further analyses with Seurat v3 (Butler et al. 2018).

#### Cross-Species Integrated Analysis

To perform cross-species comparison analysis of embryo brains between human, rhesus macaque, and tree shrew at the cellular level, we followed the steps outlined below.

##### Acquisition of Orthologous Genes Between Species

We used OrthoFinder (Emms and Kelly 2019) to determine one-to-one orthologous protein-coding genes between human (*H. sapiens*, GRCh37, Ensembl v87), rhesus macaque (*Macaca mulatta*, Mmul_8.0.1, Ensembl v97), and tree shrew (*T. belangeri*, TS_2.0 long-read assembly, http://www.treeshrewdb.org/). A total of 9,833 orthologous genes were considered for further analyses.

##### Integrated Analysis and Classification of Integrated Cell Clusters

We aligned cells of the three species together to investigate the consistency and divergence in single cells using FindIntegrationAnchors and IntegrateData functions of the Seurat R package (v3) (Butler et al. 2018). Clusters were then identified with top 20 principal components and resolution parameters of 0.5. The FindAllMarkers function in Seurat was utilized to obtain the marker genes and DEGs pairwise between species in each integrated cluster (supplementary tables S7, S8, and S19, Supplementary Material online).

After performing integrated analysis, the annotation of rhesus macaque was used as the criterion to annotate the integrated clusters. We calculated the percentage of each macaque cell type in each integrated cluster, and then the integrated cluster was annotated as the cell type with the largest percentage. Similar cell clusters showed higher correlations (supplementary fig. S2*A*, Supplementary Material online).

### RNA Extraction and Sequencing for mRNA-seq

#### RNA Extraction

Total RNA was extracted using the TRIzol method following the manufacturer's (Qiagen) protocol. OD_260/280_ ratio and RNA concentration were determined for each sample using NanoDrop (Thermo Fisher Scientific) and Qubit 2.0 (Thermo Fisher Scientific), respectively, and RNA integrity numbers (RIN) were measured using the Agilent 2100 Bioanalyzer system.

#### Library Preparation and Sequencing

Poly(A)^+^ RNA was used to construct paired-end sequencing libraries following the Illumina manual. After library construction, RNA sequencing was performed on an Illumina Novaseq 6000 sequencing platform using a 150-bp paired-end sequencing protocol with 5G data for each sample.

### mRNA-seq Data Processing

#### Reads’ Alignment and Quality Control

We newly generated 32 nonbrain transcriptomes of rhesus macaque, and 24 brain and 28 nonbrain transcriptomes of tree shrew (supplementary tables S13–S15, Supplementary Material online). Quality control was performed for raw data using fastp software (Chen et al. 2018) and Btrim64 (Kong 2011); with any reads for which N content exceeded 10% or low-quality (*Q* ≤ 5) bases exceeded 50% of the totality of bases, the corresponding paired reads were removed. Subsequently, STAR (Dobin et al. 2013) was used to map the high-quality reads to the reference genomes of rhesus macaque (*M. mulatta*, Mmul_10, Ensembl v99) and tree shrew (*T. belangeri*, TS_2.0 long-read assembly, http://www.treeshrewdb.org/), respectively, without providing junction annotation. In addition, to ensure consistency in data processing and comparability between data sets from different sources, 24 randomly selected brain transcriptomes from rhesus macaque (Li et al. 2019) were remapped to the reference genome (*M. mulatta*, Mmul_10, Ensembl v99) using STAR (Dobin et al. 2013) without providing junction annotation.

After mapping, we calculated the number and percentage of uniquely mapped reads (Dobin et al. 2013) for each sample and only retained those samples where the number of uniquely mapped reads was greater than 12 million and the percentage of uniquely mapped reads was greater than 70% (supplementary tables S13–S15, Supplementary Material online).

#### Quantification and Normalization of Gene Expression

FeatureCounts (v 2.0.0) (Liao et al. 2014) was used to count the number of reads uniquely mapped to each gene. Gene expression levels were quantified by read counting and transcripts per million (TPM).

### Analyses of PSBEGs Based on Primate and Nonprimate Transcriptomes

PSBEGs are genes that display lower expression in nonprimate brain compared with nonneural tissues, but exhibit higher expression in primate brain than in nonneural tissues. We proposed a protocol to obtain bona fide PSBEGs based on the analyses of transcriptomes of the brain and nonbrain tissues of primates (human and rhesus macaque) and a nonprimate (tree shrew) (fig. 3*A*).

#### One-to-One Orthologous Genes

We utilized OrthoFinder (Emms and Kelly 2019) software to determine one-to-one orthologous protein-coding genes between human (*H. sapiens*, GRCh38, Ensembl v99), rhesus macaque (*M. mulatta*, Mmul_10, Ensembl v99), and tree shrew (*T. belangeri*, TS_2.0 long-read assembly, http://www.treeshrewdb.org/), the annotation being based on human Ensembl v99. A total of 10,607 genes were retained for downstream analyses.

#### Cross-Species Comparative Transcriptome Analyses to Obtain Candidate PSBEGs

Cross-species comparative transcriptome analyses were difficult to perform owing to the huge differences in genetic background between the different species. Therefore, we applied a series of procedures to perform differential expression analyses between the brain and nonbrain transcriptomes of human, rhesus macaque, and tree shrew in order to obtain candidate PSBEGs.

Firstly, we only retained the counts of orthologous genes. Secondly, for each species, counts were retained only if they were expressed in more than 20% of samples. Thirdly, to ascertain the effects of covariates, we assessed the significance of the difference of each known covariate (including age, sex, and RIN) between two groups (brain and nonbrain) in human, rhesus macaque, and tree shrew using the Mann–Whitney *U* test in R program (supplementary table S20, Supplementary Material online). Finally, for each species, the DESeq2 R package (Love et al. 2014) was used to perform pairwise differential expression analysis between the brain and nonbrain tissues, with the covariates that were significantly different between the two groups being set as covariates in DESeq2 design to improve the reliability of the DEGs. The *P* values were corrected for multiple testing using the Benjamini–Hochberg procedure to estimate the FDR, and FDR < 0.05 and |log_2_ (fold change)| ≥ 1.5 were required to identify DEGs. Afterwards, we performed principal component analysis (PCA) (supplementary fig. S6, Supplementary Material online) and hierarchical clustering (supplementary fig. S7, Supplementary Material online) in R program for all samples from each species; no obvious outliers were detected.

The brain significantly highly expressed genes of human, rhesus macaque, and tree shrew, together with the brain significantly lowly expressed genes of tree shrew, were retained for further analyses (supplementary table S21, Supplementary Material online).

#### Selection Criteria for PSBEGs

We proposed a precise protocol to select bona fide PSBEGs based on the DEGs retained above (fig. 3*A*). Firstly, we took the intersection of the brain highly expressed genes of human and rhesus macaque and removed the portion that overlapped with tree shrew brain highly expressed genes, then adding human-specific brain highly expressed genes (i.e., showing no overlap with the brain highly expressed genes of rhesus macaque and tree shrew). Then, a total of 322 genes were selected for further analysis (fig. 3*A*). Secondly, we retained the genes displaying the highest expression level in human brain tissues among the various organs. Subsequently, the genes were partitioned in two separate steps: firstly, the genes that displayed the highest expression level in tree shrew nonbrain organs were identified and retained. Then we utilized the mouse gene expression matrix of brain and nonbrain organs from the ENCODE portal (https://www.encodeproject.org/) (ENCSR544BEO, ENCODE v4) (Davis et al. 2018) and identified the genes with the highest level of expression in mouse nonbrain organs, allowing the remaining 101 genes to be defined as PSBEGs (supplementary table S16, Supplementary Material online). In the second step, the portion of genes that overlapped with the tree shrew brain lowly expressed genes were retained, followed by selection of the genes with the highest expression in mouse nonbrain organs; the remaining genes were all contained in the list of PSBEGs obtained via the first method and these served as candidate PSBEGs for functional experimentation (fig. 3*A*, supplementary table S16, Supplementary Material online).

To select PSBEGs that played important roles in fetal human brain but not in fetal mouse brain, we filtered and retained PSBEGs that showed higher expression level in the SVZ, VZ, aRG, and bRG of fetal human but particularly lower expression level in fetal mouse [log_2_ (FPKM + 1) greater than 1 in more than 80% samples of fetal human and log_2_ (FPKM + 1) less than 1 in more than 80% samples of fetal mouse] (supplementary fig. S8*A*–*C*, Supplementary Material online).

### Enrichment Analyses

The online resource g: Profiler (https://biit.cs.ut.ee/gprofiler/) (Reimand et al. 2016) was used to assess the enrichment of functional categories (GO and KEGG) of genes. The *P* values were adjusted for multiple testing using the Benjamini–Hochberg procedure to estimate the FDR.

### Overlap Analyses

The R package GeneOverlap (https://github.com/shenlab-sinai/geneoverlap) was used to perform overlap analysis between different groups of genes or sequences, and the *P* values were corrected for multiple testing using Benjamini–Hochberg.

### Chromatin Interaction and Chromatin Loop Analyses

To obtain potential promoters or enhancers around the *BMP7* gene that are specifically activated in fetal human compared with fetal mouse, we firstly downloaded chromatin interaction data of brain tissues from fetal human and fetal mouse (Reilly et al. 2015). Secondly, the peaks from fetal human brain that intersected with *BMP7* extended gene regions (extending 2 Mb upstream and 2 Mb downstream of the gene region) were retained for further analyses. Then, the coordinates of the remaining peaks were converted to their corresponding coordinates in mouse by LiftOver (https://www.genome.ucsc.edu/cgi-bin/hgLiftOver, hg19 to mm9). Peaks that failed to convert, and converted peaks that showed no overlap with peaks from fetal mouse brain, were designated human specifically activated peaks. Subsequently, we downloaded chromatin loops called in human adult brain (Wang et al. 2018). Significant promoter-anchored interactions were intersected with gene promoter regions (2 kb upstream and 1 kb downstream of transcription start sites [TSS]). Then, the promoter-interacting regions were intersected with human specifically activated peaks around the *BMP7* gene as described above, the overlapping regions being attributed to potential promoters or enhancers around *BMP7*.

### IUE

#### Animals

All animal care and experimental procedures were conducted in compliance with the guidelines of the Animal Care and Use Committee (IACU) of the Kunming Institute of Zoology (approval number: SMKX-20201115-29), Chinese Academy of Sciences. Mice with an Institute of Cancer Research (ICR) background were used for IUE; the day after male and female mice were mated was demarcated as E0.5. Male and female embryos at E13.5 were used for IUE. The embryos were collected in E15.5 and E16.5 after the pregnant mice were sacrificed humanely. To test the proliferation of cortical NPCs, BrdU was injected intraperitoneally 2 h before embryo collection on E15.5 and E16.5, and cell proliferation was examined by means of BrdU incorporation.

### Experimental Constructs for IUE

The human *BMP7* plasmid was purchased from WZ Biosciences (CH807332). It was subcloned into the pCAGGS vector for IUE, and pCAGGS-EGFP plasmids were used as fluorescence tracers for IUE. The *BMP7* plasmid contained the coding sequence of *BMP7* gene along with its human-specific promoter and the EGFP reporter gene.

### Embryonic IUE

Plasmids were purified using the EndoFree Plasmid Kit (QIAGEN Plasmid plus maxi kit, 12965). About 1 μl DNA solution, including the indicated plasmids (with each plasmid 1 μg/μl) and 0.1% fast green, was injected into the medial region of the lateral ventricles of the embryonic murine brain with a glass micropipette after the pregnant mouse had been anesthetized with 3% isoflurane. The electrical pulses were delivered to the embryo under the following conditions: 25 V, 50 ms on, 950 ms off, and five pulses (BTX ECM 830). After electroporation, the uterus was returned to the abdominal cavity, followed by suture of the abdominal wall and skin. Animals were kept warm at 37 °C until recovery from anesthesia.

#### Immunofluorescence

To fix the sample, mouse embryonic brains were treated with 4% paraformaldehyde (PFA) overnight at 4°C followed by 25% sucrose incubation at 4°C. Ten-micrometer-thick coronal sections were obtained using cryostat sectioning. The following antibodies were used for immunostaining: primary antibody Ki67 (Cell Signaling, 9129) or BrdU (Abcam, ab92837); secondary Alexa Fluor 555 antibody, A-21428 or A-21437 (Invitrogen); and DAPI (DAPI: 4',6-diamidino-2-phenylindole) (Roche, 10236276001). Images were taken with a Leica TCSSP8 scanning confocal microscope.

### Luciferase Assay

The RECNEs for *BPGM* of human, rhesus macaque, and tree shrew were cloned into pGL3-promoter vector (Promega, Cat. # E1761) and cotransfected with the internal control vector pRL-TK (Promega, Cat. #E2241) into mouse cell line N2A or human cell lines HEK293T and SH-SY5Y. Cells were harvested after 48 h, and the reporter activities were analyzed with the luciferase assay system (Promega, Cat. # E1910) according to manufacturer's instructions.

### LacZ In Vivo Reporter Assay

LacZ in vivo reporter assay were performed as described in (Bi et al. 2023). The two regulatory sequences (hg19 coordinates: chr20: 55659000-55659500 and chr20: 55818275-55818595, fig. 5) of human were cloned into the Hsp68-lacZ reporter vector that was completed by the VectorBuilder platform. Transgenic mouse embryos were generated by pronuclear injection, and F0 embryos were collected at E11.5 and stained for LacZ activity. Briefly, before injection, plasmid DNA was linearized with *Xmn*I or *Ahd*I, followed by purification with the GelExtraction Kit (Omega, D2500-01). The DNA was diluted to a final concentration of 2 ng/μl and injected into zygotes, and HCZB medium was used for injection. The injected zygotes were cultured in KSOM culture medium at 37 °C under 5% CO_2_ and 5% O_2_ in air for approximately 15 h. Thereafter, zygotes were transferred into the uteruses of ICR females. Embryos were harvested at E11.5 in cold PBS, followed by 30 min of incubation with 4% PFA. The embryos were washed three times for 30 min with embryo wash buffer (2 mm MgCl_2_; 0.01% deoxycholate; 0.02% NP-40; and 0.1 m PBS buffer, pH 7.3). LacZ activity was detected by incubating with freshly made staining solution (1 mg/ml X-gal [sigma, V900468]; 5 mm potassium ferrocyanide; 5 mm potassium ferricyanide; and 5 mm EGTA, pH 7.5, in wash buffer) at 37 °C for a few hours to overnight until the desired staining was achieved. Following staining, embryos were washed in LacZ buffer and stored at 4 °C in 4% PFA in PBS. Finally, embryos were imaged using a Nikon SMZ18 stereo microscope.

## Supplementary Material

msad173_Supplementary_Data

## Data Availability

The raw RNA-seq data of rhesus macaque and tree shrew are deposited in the NCBI Sequence Read Archive under BioProject ID PRJNA761017. The scRNA-seq data of tree shrews are available through the Gene Expression Omnibus (GEO accession number: GSE233757).
