# Supplementary material for: Integrative Omics Reveals Rapidly Evolving Regulatory Sequences Driving Primate Brain Evolution
Source: Mol Biol Evol. 2023 Jul 26;40(8):msad173. doi: 10.1093/molbev/msad173 (PMC10404817; doi:10.1093/molbev/msad173)
Supplement: msad173_Supplementary_Data [file msad173_supplementary_data.zip › Supplementary materials.docx]

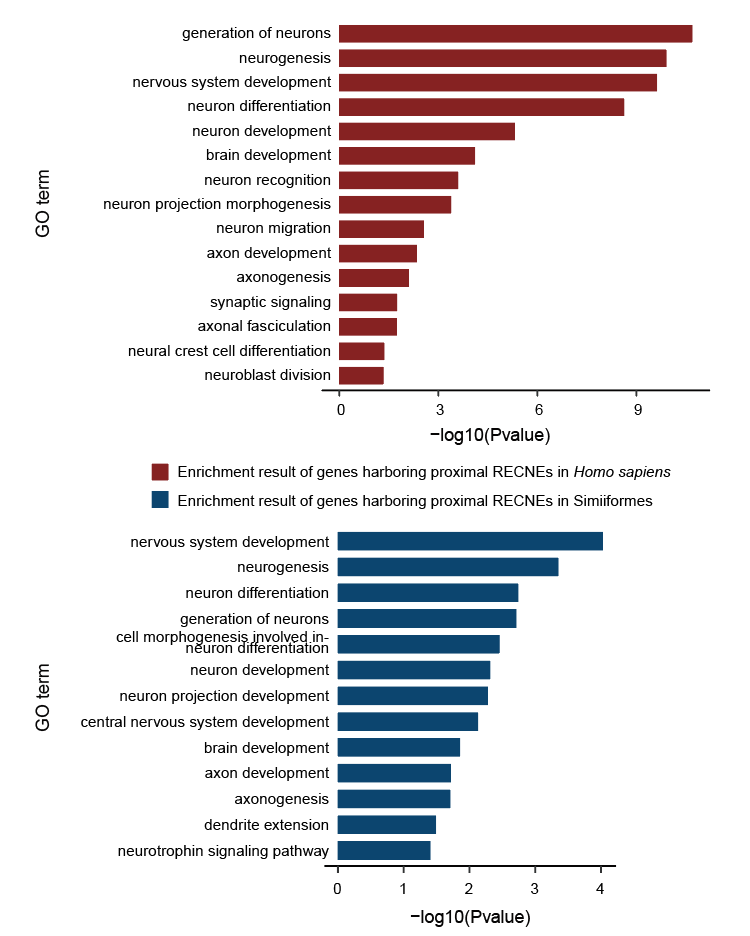


**Fig. S1.** Functional enrichment of genes harboring proximal RECNEs in the lineages of *Homo sapiens* (above) and the ancestor of the Simiiformes (below). Gene Ontology (GO) enrichments were adjusted for multiple comparisons (FDR < 0.05).


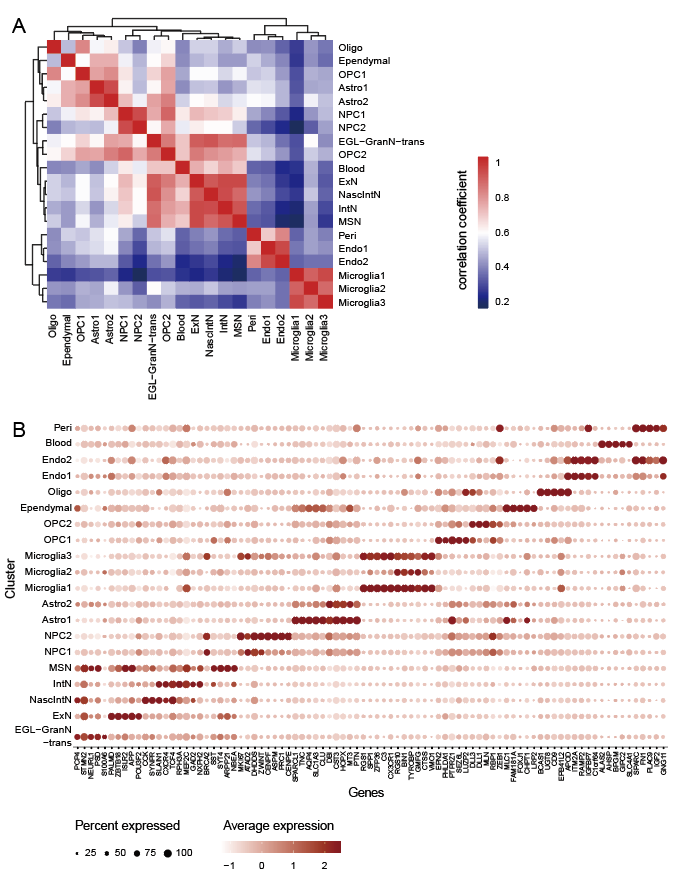


**Fig. S2. (A)** Dendrogram and heatmap show the Pearson correlation coefficients between pairs of identified cell clusters in the integrated analysis. The color bar corresponds to the correlation coefficient value. **(B)** Dot plot represents expression levels of top 5 marker genes across each integrated cluster. Dot size represents the proportion of cells expressing the marker of interest. Color bar indicates the mean expression level within expressing cells. NPC, neuronal progenitor cell; NascIntN, nascent inhibitory neuron; ExN, excitatory neuron; Astro, astrocyte; OPC, oligodendrocyte progenitor cell; Oligo, oligodendrocyte; Endo, endothelial; Peri, pericyte; EGL-GranN-trans, external granular layer transformed granule neuron; IntN, inhibitory neuron; MSN, medium spiny neuron.


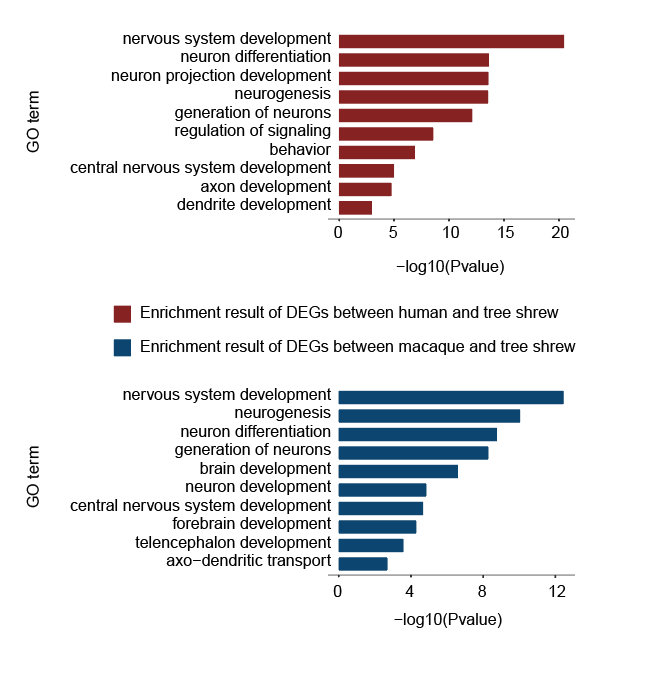


**Fig. S3.** Functional enrichment of differentially expressed genes (DEGs) between human and tree shrew (above), between rhesus macaque and tree shrew (below) in the cell cluster annotated as external granular layer transformed granule neurons (EGL-GranN-trans). Gene Ontology (GO) enrichments were adjusted for multiple comparisons (FDR < 0.05).


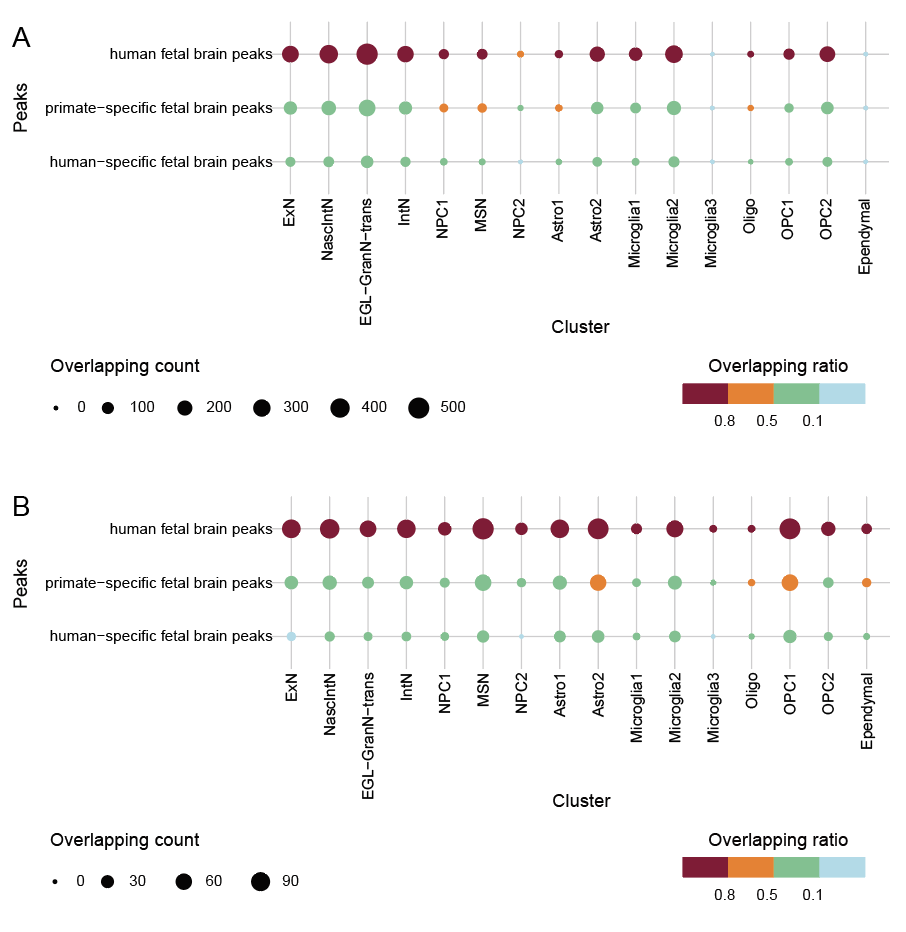


**Fig. S4. (A)** The overlap results between differentially expressed genes (DEGs, between human and tree shrew) of each cell cluster from the integrated analysis and genes harboring activated peaks in human fetal brain, only in primate fetal brain (primate: human and rhesus macaque; compared with mouse) and only in human fetal brain (compared with rhesus macaque and mouse), respectively. **(B)** The overlap results between differentially expressed genes (DEGs, between rhesus macaque and tree shrew) of each cell cluster from the integrated analysis and genes harboring activated peaks in human fetal brain, only in primate fetal brain (primate: human and rhesus macaque; compared with mouse) and only in human fetal brain (compared with rhesus macaque and mouse), respectively. Counts of overlapping genes are plotted (with number indicated by the dot size), the ratio of overlap being denoted by color (the ratio of intersection over the total number of DEGs in each cell cluster was used to depict the extent of overlap). NPC, neuronal progenitor cell; NascIntN, nascent inhibitory neuron; ExN, excitatory neuron; Astro, astrocyte; OPC, oligodendrocyte progenitor cell; Oligo, oligodendrocyte; Endo, endothelial; Peri, pericyte; EGL-GranN-trans, external granular layer transformed granule neuron; IntN, inhibitory neuron; MSN, medium spiny neuron.


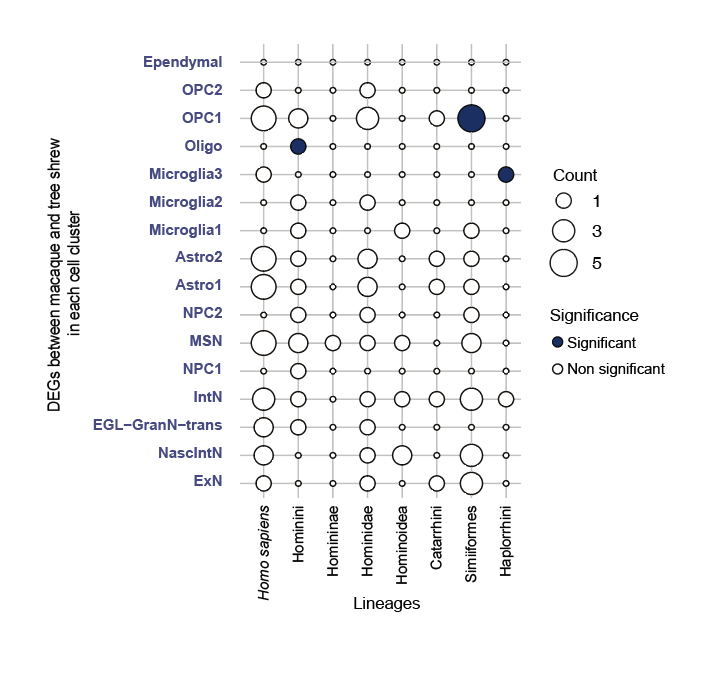


**Fig. S5.** The overlap results between genes harboring proximal RECNEs in each primate lineage and DEGs between rhesus macaque and tree shrew. The significance of the overlap was measured by Fisher’s exact test, and the p values were corrected for multiple testing using Benjamini-Hochberg. Counts of overlapping genes are plotted (with the number indicated by dot size), the significance of the overlap being denoted by color (dark blue, Significant; white, Non-significant). NPC, neuronal progenitor cell; NascInN, nascent inhibitory neuron; ExN, excitatory neuron; Astro, astrocyte; OPC, oligodendrocyte progenitor cell; Oligo, oligodendrocyte; Endo, endothelial; Peri, pericyte; EGL-GranN-trans, external granular layer transformed granule neuron; IntN, inhibitory neuron; MSN, medium spiny neuron.


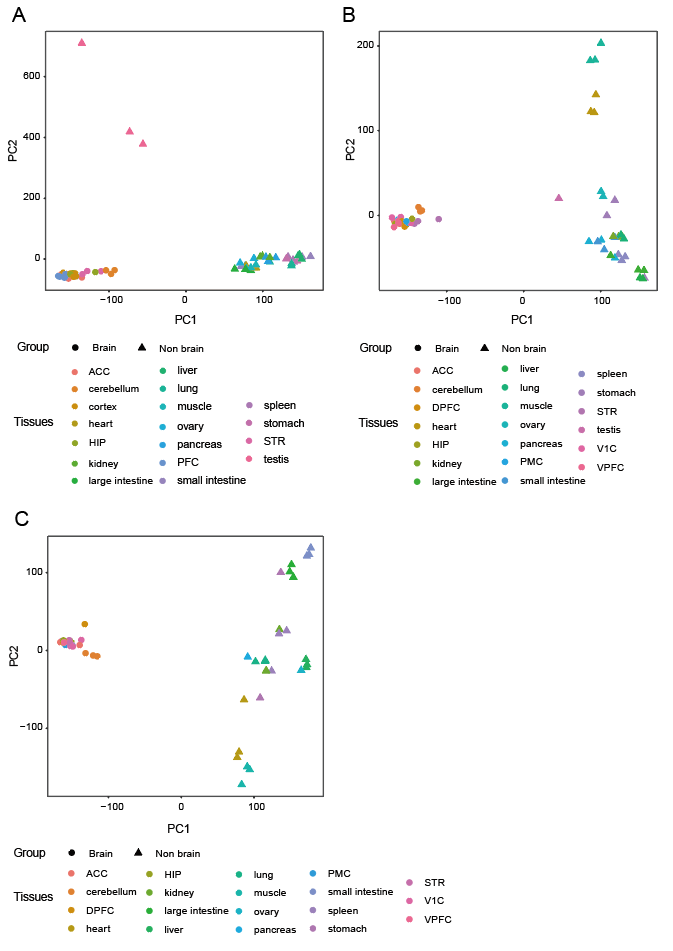


**Fig. S6.** Principal component analysis (PCA) showing transcriptional divergence of brain tissues and non-neural tissues from human **(A)**, rhesus macaque **(B)** and tree shrew **(C)**, respectively. ACC, Anterior cingulate cortex; HIP: Hippocampus; PFC, Frontal cortex; STR, Striatum; DPFC, Dorsolateral prefrontal cortex; PMC, Premotor cortex; V1C, Primary visual cortex; VPFC, Ventral lateral prefrontal cortex


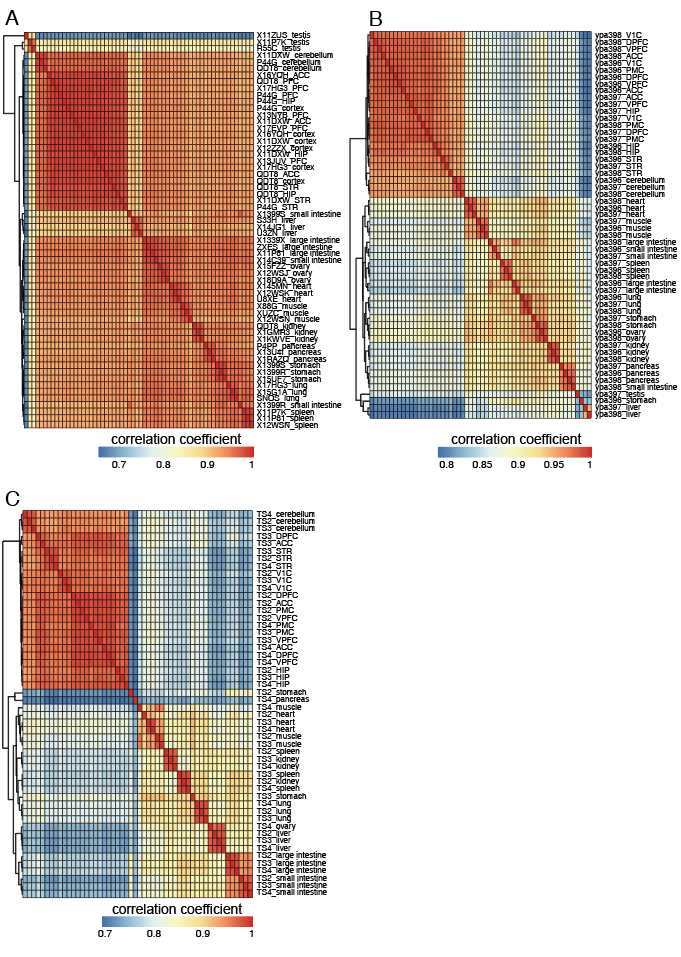


**Fig. S7.** Hierarchical clustering of RNA samples showing distinct clusters of brain tissues and non-neural tissues from human **(A)**, rhesus macaque **(B)** and tree shrew **(C)**. The naming format of RNA samples was “individual name_tissue name”. ACC, Anterior cingulate cortex; HIP: Hippocampus; PFC, Frontal cortex; STR, Striatum; DPFC, Dorsolateral prefrontal cortex; PMC, Premotor cortex; V1C, Primary visual cortex; VPFC, Ventral lateral prefrontal cortex.


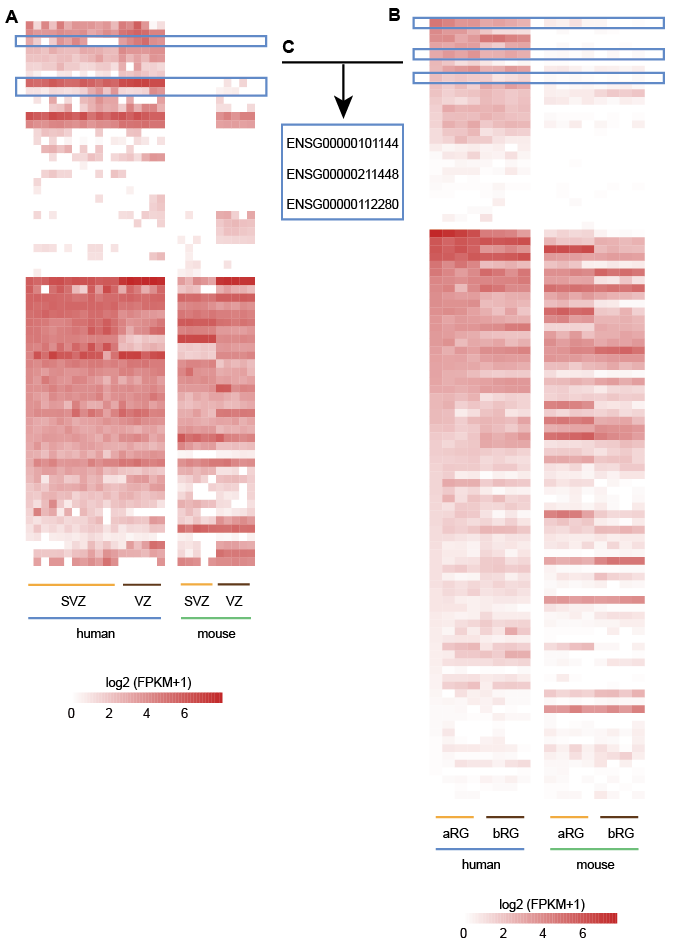


**Fig. S8.** Heatmap depicting the expression profile of PSBEGs in subventricular zone (SVZ), ventricular zone (VZ) brain regions (**A**) and apical radial glia (aRG), basal radial glia (bRG) cells (**B**) from fetal human and fetal mouse, respectively; transcriptome data were from ([Fietz, et al. 2012](#_ENREF_1); [Florio, et al. 2015](#_ENREF_2)). Three genes [ENSG00000101144 (*BMP7*), ENSG00000211448 (*DIO2*), and ENSG00000112280 (*COL9A1*)] that showed the expected pattern in both SVZ, VZ and aRG, bRG of fetal human and fetal mouse are shown in (**C)**. Regions and cell types are distinguished by color (yellow, SVZ, aRG; brown, VZ, bRG), species are distinguished by color (blue, human; green, mouse).


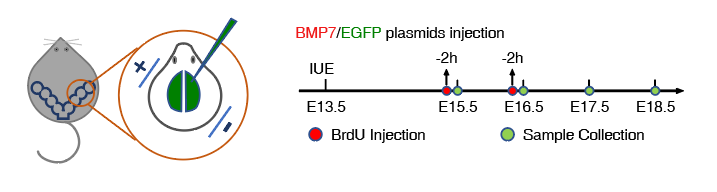
**Fig. S9.** Schematic diagram of *in utero* electroporation (IUE) following BrdU injection and sample collection at designated time points. Enhanced green fluorescent protein (EGFP) only or together with *BMP7*-containing plasmids were electroporated into E13.5 embryos and pregnant mice were killed at E15.5, E16.5, E17.5 and E18.5 time points, respectively. BrdU (50 mg/kg body weight, i.p.) was injected at E15.5 and E16.5 before sample collection.


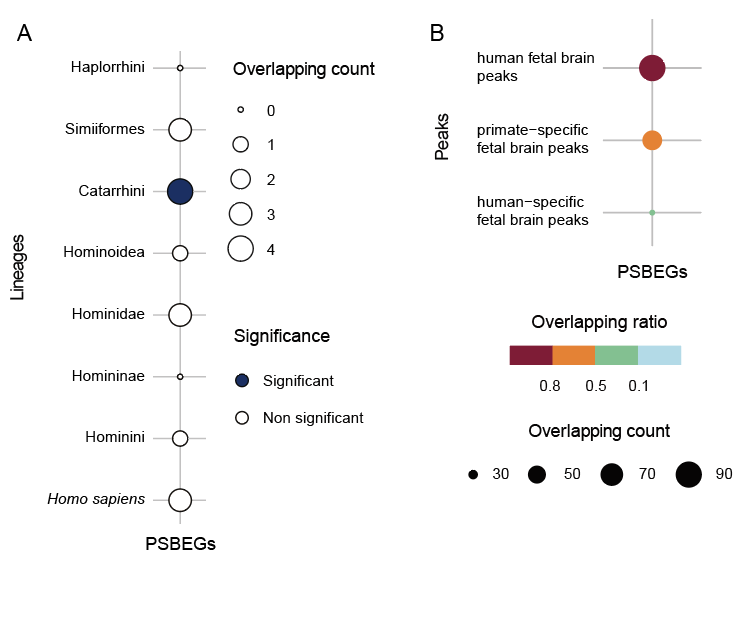


**Fig. S10.** **(A)** The overlap results between primate-specific brain-biased genes (PSBEGs) and genes harboring proximal RECNEs in different primate lineages. The significance of the overlap was measured by the Fisher’s exact test, and the p values were corrected for multiple testing using Benjamini-Hochberg. Counts of overlapping genes are plotted (with the number indicated by the size of dots). Significance is denoted by color (dark blue, Significant; white, Non-significant). **(B)** The overlap results between PSBEGs and genes harboring activated peaks in human fetal brain, only in primate fetal brain (primate: human and rhesus macaque; compared with mouse) and only in human fetal brain (compared with rhesus macaque and mouse), respectively. Counts of overlapping genes are plotted (with number indicated by the dot size), the ratio of overlap is denoted by color (the ratio of intersection over the total number of PSBEGs was used to depict the extent of overlap).


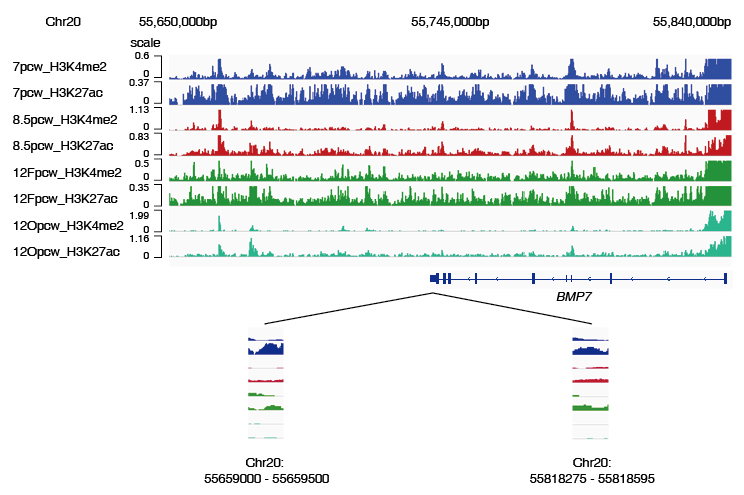


**Fig. S11.** IGV screenshots of eight CHIP-seq samples from fetal human brains in two human specifically activated promoters or enhancers around the *BMP7* gene. Below, expanded view of the two human specifically activated promoters or enhancers. The chromatin interaction data of fetal human brain were obtained from ([Reilly, et al. 2015](#_ENREF_4)).

**
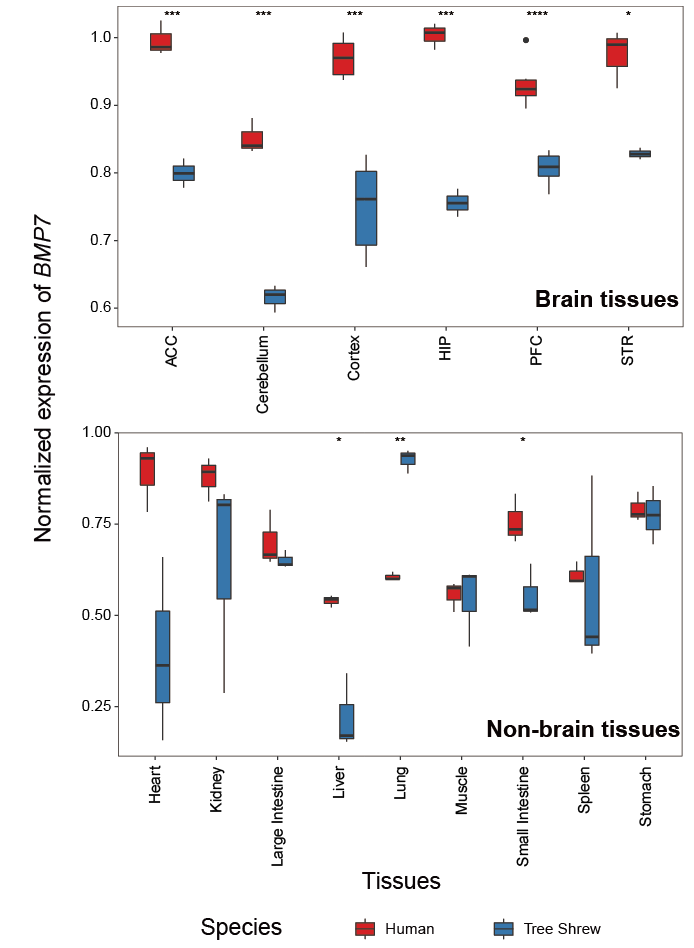
**

**Fig. S12.** Boxplot depicting the normalized expression of *BMP7* in brain (above) and non-neural (below) tissues from human (filled in red) and tree shrew (filled in blue). The normalized expression of *BMP7* in each species was calculated by the median of ratios method in DEseq2 ([Love, et al. 2014](#_ENREF_3)); once the sequencing depth and RNA composition had been taken into account, the normalized gene counts could be compared between samples. Then, for each tissue in each species, we calculated the average expression level of the top 10000 genes, then the normalized expression level of each gene divided by the average expression level in each tissue in each species. Data in each group were analyzed using an Unpaired t test with Welch's correction. * p <0.05; ** p<0.01; *** p<0.001. ACC, Anterior cingulate cortex; HIP: Hippocampus; PFC, Frontal Cortex; STR, Striatum; DPFC, Dorsolateral prefrontal cortex; PMC, Premotor cortex; V1C, Primary visual cortex; VPFC, Ventral lateral prefrontal cortex.

**Supplementary Tables**

Supplementary tables are provided in one Microsoft Excel file.

**References**

Fietz SA, Lachmann R, Brandl H, Kircher M, Samusik N, Schroder R, Lakshmanaperumal N, Henry I, Vogt J, Riehn A, et al. 2012. Transcriptomes of germinal zones of human and mouse fetal neocortex suggest a role of extracellular matrix in progenitor self-renewal. Proc Natl Acad Sci U S A 109:11836-11841.

Florio M, Albert M, Taverna E, Namba T, Brandl H, Lewitus E, Haffner C, Sykes A, Wong FK, Peters J, et al. 2015. Human-specific gene *ARHGAP11B* promotes basal progenitor amplification and neocortex expansion. Science 347:1465-1470.

Love MI, Huber W, Anders S. 2014. Moderated estimation of fold change and dispersion for RNA-seq data with DESeq2. Genome Biol 15:550.

Reilly SK, Yin J, Ayoub AE, Emera D, Leng J, Cotney J, Sarro R, Rakic P, Noonan JP. 2015. Evolutionary changes in promoter and enhancer activity during human corticogenesis. Science 347:1155-1159.
